# The Effect of Ceramic Membranes’ Structure on the Oil and Ions Removal in Pre-Treatment of the Desalter Unit Wastewater

**DOI:** 10.3390/membranes12010059

**Published:** 2021-12-31

**Authors:** Yaser Rasouli, Mohammad Mehdi Parivazh, Mohsen Abbasi, Mohammad Akrami

**Affiliations:** 1 Department of Civil, Geological & Mining Engineering, Ecole Polytechnique de Montreal, 2900 Boulevard Edouard-Montpetit, Montreal, QC H3T 1J4, Canada; yaser.rasouli@polymtl.ca; 2 Department of Chemical Engineering, Amirkabir University of Technology (Tehran Polytechnic), Tehran P.O. Box 15875-4413, Iran; m.parivazh@gmail.com; 3 Department of Chemical Engineering, Faculty of Petroleum, Gas and Petrochemical Engineering, Persian Gulf University, Bushehr P.O. Box 75169-13798, Iran; 4 Department of Engineering, University of Exeter, Exeter EX4 4QF, UK

**Keywords:** ceramic membrane, oily wastewater, desalter unit, microfiltration, salt/oil rejection, permeation flux

## Abstract

Salts, organic materials, and hazardous materials can be found regularly in the effluent from a desalter unit of crude oil. These materials should be separated from the wastewater. Four kinds of inexpensive and innovative ceramic microfiltration membranes (mullite, mullite-alumina (MA 50%), mullite-alumina-zeolite (MAZ 20%), and mullite-zeolite (MZ 40%)) were synthesized in this research using locally available inexpensive raw materials such as kaolin clay, natural zeolite, and alpha-alumina powders. Analyses carried out on the membranes include XRD, SEM, void fraction, the average diameter of the pores, and the ability to withstand mechanical stress. Effluent from the desalter unit was synthesized in the laboratory using the salts most present in the desalter wastewater (NaCl, MgCl_2_, and CaCl_2_) and crude oil. This synthesized wastewater was treated with prepared ceramic membranes. It was discovered that different salt concentrations (0, 5000, 25,000, 50,000, 75,000, and 100,000 mg L^−1^) affected the permeate flux (PF), oil rejection, and ion rejection by the membrane. Results showed that in a lower concentration of salts (5000 and 25,000 mg L^−1^), PF of all types of ceramic membranes was increased significantly, while in the higher concentration, PF declined due to polarization concentration and high fouling effects. Oil and ion rejection was increased slightly by increasing salt dosage in wastewater due to higher ionic strength. Monovalent (Na^+^) and multivalent (Ca^2+^ and Mg^2+^) ion rejection was reported about 5 to 13%, and 23 to 40% respectively. Oil rejection varied from 96.2 to 99.2%.

## 1. Introduction

**Figure 1:**
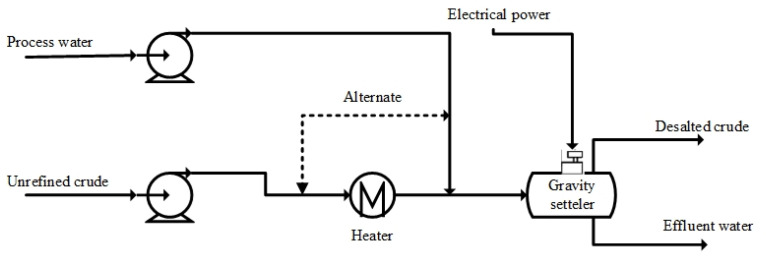
Schematic diagram of an electrostatic desalting unit.

Crude oil desalting and dewatering upstream from the crude distillation unit is a critical process activity for removing unwanted components from crude oil before it enters any of the primary unit operations [1]. Due to constantly shifting process parameters, running a desalting system can be extremely difficult. The desalters’ primary purpose is to remove salt and water from crude oil. The desalter effluent, or under carry, is a mix of mud wash and produced water, as well as diluted and desalinated salty wash water and other contaminated saline oily wastewater [2]. In order to utilize this wastewater in the industry, firewater, and agriculture, it must be treated. The components of oily wastewater are complex, including high salinity, types of surfactants, and other organic matters, such as emulsified oils [3,4]. Heavy metals dissolved in water are common in crude oil, as are other impurities such as inorganic salts, suspended particles, and water. Desalting units get rid of these pollutants [5,6]. As illustrated in Table 1, NaCl, MgCl_2_, and CaCl_2_ constitute the most prevalent salts in the wastewater of the desalter unit [3]. The presence of water and salts in the crude oil causes corrosion in the piping and other process equipment, temperature, pressure drops in the system, and many other problems in refining crude oils [7]. Therefore, when salt concentration exceeds 10 PTB, desalination of crude oil before the refining process is inevitable [8,9]. On industrial scales, desalination of crude oil in desalter units is carried out by two main methods. Chemical and electrostatic separations are the most common methods of desalting crude oil [7]. Chemical desalting involves adding water and chemical surfactants (demulsifiers) to crude oil, followed by heating to cause salts and other contaminants to dissolve into or attach to the water [10,11]. The concentration of water droplets at the bottom of the settling tank is achieved using high-voltage electrostatic charges in electrical desalination [12,13,14]. A schematic diagram of electrostatic desalting is shown in Figure 1. During the heating process, the raw material crude oil is heated to temperatures ranging from 65 to 176 °C to decrease the viscousness and tendency of liquid surfaces, allowing for better water mixing and separation. The temperature is constrained by the crude-oil feedstock’s vapor pressure. Additional chemicals may be added to either method. Corrosion-reduction techniques frequently call for the use of ammonia. To make the water wash more alkaline, caustic or acid solution can be added. The bottom of the settling tank releases waste and impurities into the wastewater treatment plant, which is the objective of this investigation. On top of the settling tanks is where desalted crude oil is constantly being drawn down to be used in a fractionating tower for further processing [7,10,11,12,13,14,15,16].

Ceramic membranes are a form of synthetic membrane constructed entirely of inorganic compounds (such as alumina, titania, zirconia oxides, silicon carbide, or some glassy materials). They are employed in the liquid filtration membrane processes. Ceramic membranes have been around for a long time, and they are utilized in a wide variety of applications due to their various benefits: enduring stability in extreme conditions of heat and pressure, a high degree of chemical resistance, high tensile strength, exceptional longevity, and antifouling characteristics. Filtration with ceramics is a mild, highly selective process without phase transformation. Running costs are limited by closed production cycles and continuous processes. The disadvantages are their high weight and the considerable production costs of ceramic components. However, the latter is generally compensated by a long service life [17].

Produced water and desalter effluent are similar to each other in many ways. They have high oil and organic matter, salt, and suspended solids content. The difference between produced water and desalter effluent is referred to as the amount of these hazardous contents in which desalter effluent contains a higher amount of these contents. Produced water was treated by using membrane distillation [18], forward osmosis [19], ceramic microfiltration membrane [20], polymeric ultrafiltration membrane [21], reverse osmoses [22], nanofiltration [23], etc.

Studies are primarily focused on treating the produced water by different methods [24,25,26,27], whereas research into the treatment of effluent from desalter units employing membrane technologies is limited. In an experimental study conducted by Bijani et al. [28], untreated wastewater from the desalting of crude oil was treated with reverse osmosis membranes. Reverse osmosis-process refined water was found to be an appropriate and cost-effective injection option for both onshore and offshore fields in part because of the low salt content, the ion concentrations involved in scaling formation, and the proper pH. Dadari et al. [5] used an innovative synthetic high-performance nanofiltration membrane composed of adipate ferroxane nanoparticles for desalter effluent treatment. In order to analyze, model, and optimize the process, three independent variables were selected: feed concentration, transmembrane pressure, and nanoparticle concentration. The results showed that nanofiltration performance for desalter effluent was optimal at a 0.5% (wt) nanoparticle concentration. In all experiments, COD removal and permeation flux were reported at 71.4–93.5% and 3.1–22.5 kg/m^2^ h, respectively. Nizam [29] proposed a new treatment system based on absorption and reverse osmosis membrane for treating desalter’s produced water. Various operating conditions (transmembrane pressure, feed concentration, feed pH) were studied in order to achieve the optimal removal of salts and impurities from the produced water. The membrane removed more than 98% of TSS, salinity, and salt ions in addition to removing more than 95% of COD. Norouzbahari et al. [6] treated crude oil desalter effluent from a Tehran oil refinery using a hybrid UF/RO membrane separation process. In UF pre-treatment, researchers looked at the impact of operational parameters such as the pressure drop across the membrane barrier and the linear velocity of the flow tangential to the membrane surface. The results of the experiments indicated that the UF membrane eliminated more than 75% of the oil and RO removed more than 95% of TDS from the desalter effluent. The conditions of preparation of low-cost membranes from clay and zeolite were studied by different scholars [30,31,32]. Materials and procedures for producing low-cost membranes from raw materials such as clays and zeolites are discussed by Abdullayev et al. [33]. Natural zeolites are ground, shaped, and sintered to provide bulk materials with the required aluminosilicate phases for membrane fabrication. The sintering temperatures of kaoline and zeolite ranged from 850–1550 °C and 800–900 °C, respectively. To remove Cr(VI) from surface water, ceramic membranes comprised of clay, perlite, and iron were created by Angel et al. Membranes with a diameter of 7 cm and a thickness of 1 cm were sintered at 950 °C with different amounts of iron filings in the clay-perlite matrix (0, 0.5, 1, and 1.5 wt.%) [34].

In this study, four distinct kinds of economical and innovative ceramic microfiltration membranes were created utilizing kaolin clay, alpha-alumina powder, and natural zeolite as initial ingredients by an extrusion method and called mullite, MA 50%, MAZ 20%, and MZ 40% membranes. In this study, the aim is to evaluate the efficacy of prepared membranes for desalter effluent filtration. Effects of salt concentrations and the membrane type on the salt/oil rejection and the PF are studied. Synthesis and applications of such inexpensive and high-performance ceramic membranes with the different compositions of kaolin and adsorbents, as a pre-treatment step for other processes such as RO, would be very interesting and highly beneficial to fill gaps in the literature.

## 2. Material & Methods

### 2.1. Materials

The Merck Company (Darmstadt, Germany) provided all of the chemical materials used, such as sodium chloride (NaCl > 99.5% purity), calcium chloride (CaCl_2_ > 98% purity), magnesium chloride (MgCl_2_ > 99.5% purity), and Tritton X-100 (C_14_H_22_O(C_2_H_4_O)n(n = _9–10_) > 99.5%). Iran’s Gachsaran oil field provided the crude oil used in this project. The Zenooz mine in Tabriz, Iran, provided the kaolin clay used in this project. 99.6% pure alpha-alumina powder with an average particle size of 75 µm is provided from the Iranian alumina Company (Jajarm, Iran). Natural zeolite powder is supplied from Semnan mines, Iran. Table 2 contains chemical analyses of kaolin and natural zeolite.

### 2.2. Ceramic Membranes Fabrication

#### 2.2.1. Mullite Membrane

**Figure 2:**
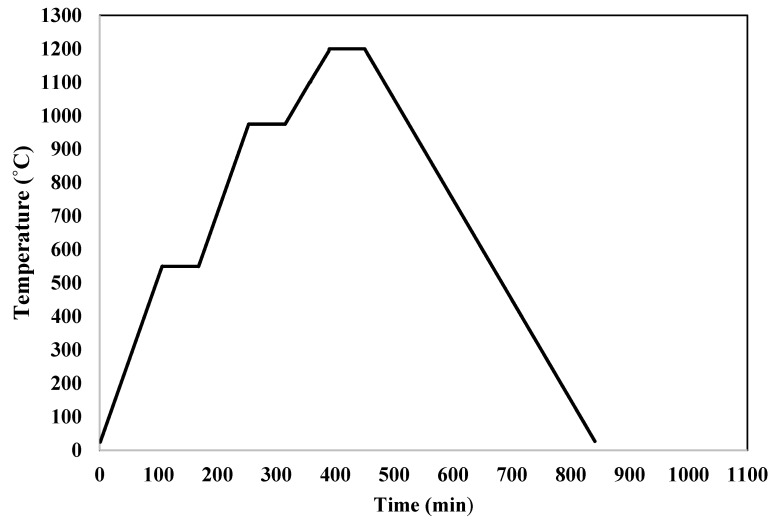
Variation of temperature over time during sintering membranes.

Four distinct kinds of innovative and inexpensive ceramic microfiltration membranes are created using the extrusion technique utilizing locally available powders. In order to make mullite membranes, 69 wt.% kaolin clay is combined with 31 wt.% distilled water. Extrusion was used to make the kaolin clay/distilled water mixture after mixing well for 2 h using a high-power mechanical mixer. A tube-shaped membrane with a 10 mm inner diameter, 14 mm outside diameter, and a length of 10 cm was created. At room temperature, prepared membranes are allowed to dry completely for twenty-four hours and then sintered in the programmable furnace (1200C MINI LAB ELECTRIC, T-Long, Zhengzhou, China). The rate of change in temperature from ambient to 550 °C was 5 °C per minute. A constant 550 °C temperature was then applied for an hour. The temperature was raised at a rate of 5 °C per minute from 550 to 975 °C. After an hour, the temperature was set at 975 °C and then increased at a rate of 3 °C per minute until it reached 1200 °C and kept fixed at 1200 °C for an hour. Figure 2 shows the temperature gradient during sintering membranes. After cooling in the furnace for 8 h, sintered membranes were taken out to avoid breaking due to sudden temperature changes. Temperatures and times for calcination are appropriate when clay transforms into mullite and free silica. Calcinating at temperatures of about 1250 °C yielded promising results [35]. In sintering temperature 550 °C, an Endothermic chemical reaction resulted in metakaolin being produced from the kaolinite phase (Equation (1)).

(1) $$2{\mathrm{Al}}_{2}{\mathrm{Si}}_{2}O_{5}(\mathrm{OH}{)}_{4}\to 2{\mathrm{Al}}_{2}{\mathrm{Si}}_{2}O_{7}+4H_{2}O$$

In a sintering temperature of 1050 °C, under the following reaction, kaolin transforms into mullite phase and free silica.

(2) $$3({\mathrm{Al}}_{2}O_{3}\cdot 2{\mathrm{SiO}}_{2})\to 3{\mathrm{Al}}_{2}O_{3}\cdot 2{\mathrm{SiO}}_{2}+4\mathrm{Si}$$

**Figure 3:**
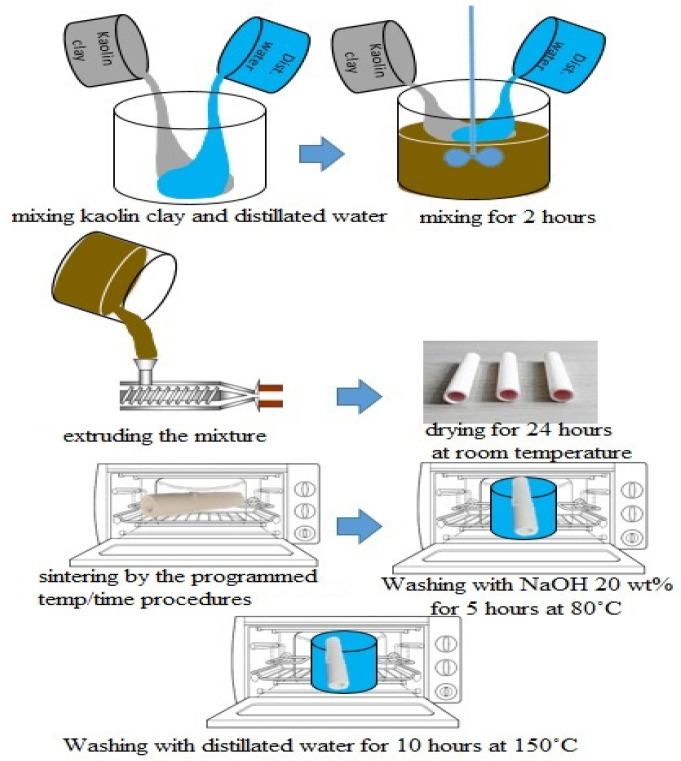
Schematic representation of mullite membrane fabrication.

To make the membranes more porous, an alkali solution with a high pH value (20 wt.% NaOH) was used to wash the free silica in an oven for five hours at 80 °C. In the end, any remaining NaOH was washed away using distilled water in a 150 °C oven for ten hours. Schematic representation of mullite membrane preparation is shown in Figure 3.

#### 2.2.2. MA Membrane

For the preparation of MA membranes, distilled water and 50% kaolin clay were combined with 50 wt.% alpha-alumina powder. A crushing machine triturated huge kaolin samples into fine particles with a filter size of 200 mesh before combining. This mixture was mixed well for 2 h by using a mixer. Then the mixture was extruded and sintered like mullite membranes. Sintering temperature and other procedures for preparing MA membranes were similar to mullite membranes.

#### 2.2.3. MAZ Membrane

Natural zeolite samples were pulverized by the crushing machine and then collected as fine powders after passing through the 200-mesh sieve. MAZ membranes were prepared by adding 30 wt.% alpha-alumina powder and 20 wt.% natural zeolite to a combination of 50 wt.% kaolin and distilled water; then, this mixture was mixed well to homogenize it. Extrusion and sintering procedures were done in ways similar to mullite and MA membrane preparation.

#### 2.2.4. MZ Membrane

MZ membranes were prepared by adding 40 wt.% natural zeolite powder to a combination of 60 wt.% kaolin clay and distilled water, followed by extrusion and sintering processes like other membranes. It should be noted that during the synthesis of the membranes, there was no additive addition; only kaolin, alumina, and natural zeolite with the mentioned compositions. Furthermore, the percentage choice of the different materials was based on the authors’ previous study [36], in which among the tested membranes, Mullite, MA with 50 wt.% alumina, MAZ with 30 wt.% alumina and 20 wt.% natural zeolite, and MZ with 40 wt.% natural zeolite are the best membranes in terms of flux and contaminants removal.

### 2.3. Characterization Techniques

We utilized scanning electron microscope equipment (SEM, Tescan Vega 3 manufactured in the Czech Republic) with a 20 kV accelerating voltage to characterize the surface and cross-section morphology of four types of the fabricated membranes (mullite, MA 50%, MAZ 20%, and MZ 40%). A crushing device was used to triturate large membrane samples into smaller samples. To boost specific conductance, these tiny samples were subsequently coated with a thin film of gold for better analysis in the microscope.

After sintering the membranes, an X-ray diffraction apparatus (Philips PW1800 with Cu Kα radiation, Amsterdam, The Netherlands) was used to identify the produced phases in their structure. The accelerating voltage and the applied currents were 40 kV and 40 mA, respectively. For XRD analysis, membranes samples were cleaned and pulverized by using a crushing machine in order to form fine powders.

The Guerout–Elford–Ferry Equation (Equation (3)) was used to measure the average membrane pore radius (r_m_). For each TMP (0.25–4 bar), we determined the pure water flux across membranes. The slope of the linear Equation obtained by graphing TMP against pure water flux was used to determine r_m_.

(3) $$r_{m}=\sqrt{\frac{\left(2.9-1.75\varepsilon \right)\times 8\mu L\times \mathrm{PF}}{\varepsilon \times \mathrm{TMP}}}$$

where ε is the void volume fraction of the membrane (%), μ is the viscosity of water (8.9 × 10^−4^ Pa·s) at its operating temperature, L is the thickness of the membrane (m), and PF stands for pure water flux when a certain TMP is used [37,38].

For measuring oil emulsion droplet size, a dynamic light scattering particle size analysis was used (Microtrac, MN402-NS-0000-0000-000-4M, Montgomeryville, PA, USA). Analyses were performed immediately after fabrication of the emulsion sample (1 mL). Particle sizes between 0.3 and 10,000 nm were measured using the instrument. The emulsion’s mean oil droplet size distribution was determined using Equation [39,40]:

(4) $$d_{\mathrm{average}}={\left(\frac{\sum_{i=1}^{n}n_{i}d_{i}^{2}}{\sum_{i=1}^{n}n_{i}}\right)}^{0.5}$$

When viewing this Equation, n is the proportion of volume in a unit of time, d_average_ is the average diameter of oil droplets, and d_i_ is the diameter corresponding to each oil droplet.

The measurement of the mechanical resistance of the membrane was chosen based on a SANTAM, STM-150 universal testing device with 150 KN capacity, total grips distance 690 mm, dimensions (Width × Depth × Height (mm)) 1070 × 700 × 2430, and 200 V 50–60 Hz power using the three-point bend test with ASTM C1505-01 [41]. Industrial quality control labs and research institutes have long relied on the SANTAM universal testing equipment. Many different materials may be tested with this equipment. Metals and ceramics are only two examples.

The measurement of membrane porosity was done by comparing the dry and wet weight of the membrane. The membrane kept in distilled water for 3 h was weighed after wiping off superficial water (W_2_). The wet membrane was then heated in an electrical oven set to 150 °C for 5 h before the dry mass was determined (W_1_). The porosity of the membrane was determined as follows using the mass of the wet and dry samples [42,43]:

(5) $$\mathrm{porosity}=\left(\frac{W_{1}-W_{2}}{\rho_{M}V_{M}}\right)\times 100$$

The dry and wet membrane masses and volumes are represented by W_2_, W_1_, and V_M_, respectively, while the water density at the temperature of the experiment is represented by ρ_m_.

### 2.4. Preparing Desalter Unit Wastewater

To produce desalter unit wastewater in the lab, we combined crude oil, distilled water, Tritton X-100 (an emulsifier agent with a crude oil content of only 0.01 wt.%), and three kinds of salts (NaCl 75 wt.%, CaCl_2_ 15 wt.%, and MgCl_2_ 10 wt.%) for a total concentration of 0, 5000, 25,000, 50,000, 75,000, and 100,000 mg L^−1^. Table 3 summarizes the analysis of the physical and chemical properties of crude oil. The combination was then subjected to a 30-min high-speed homogenization process (using a Wise Mix Homogenizer HG 15) at 19,000 rpm. The concentration of oil in the synthetic wastewater in all tests was fixed at 1000 mg L^−1^.

### 2.5. Experimental Setup & Operations

**Figure 4:**
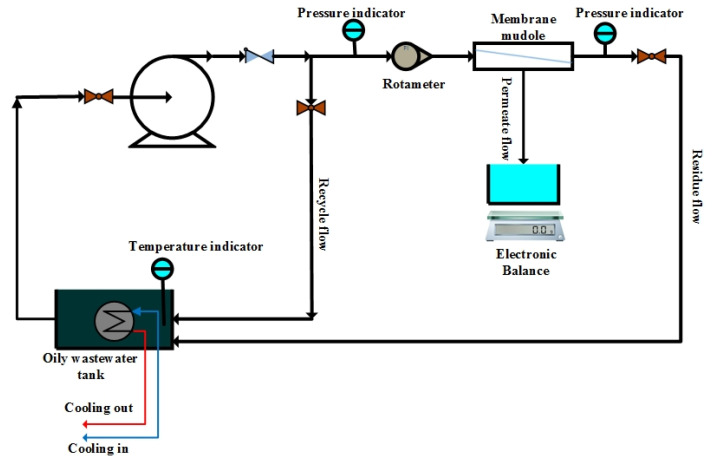
Experimental setup used for investigation of ceramic membrane performance during desalter wastewater treatment.

Figure 4 depicts the experimental setup. We intended our setup to be as simple as possible, and we had complete control over all operational parameters, including temperature, transmembrane pressure, and cross-flow velocity (CFV). Previously, various operational parameters for mullite membranes used to treat synthetic wastewaters had been studied concerning their impacts on the PF, resistance to fouling substance buildup, and refusal of oil. These parameters included TMP (0.5–4 bar) and CFV (0–2 m per second), as well as T (15–55 °C) [44]. Consequently, these optimal operating parameters (TMP = 3 bar, CFV = 1.5 m s^−1^, and T = 35 °C) were used in this research. A heater and a cooling system were installed in each tank to regulate the fluid temperature. For all fabricated ceramic microfiltration membranes, PF and a rejection of oil and salt were achieved.

PF is defined by Equation (6) as the volume (V) of accumulated permeate in the unit of time (t) and the membrane surface area (A):

(6) $$\mathrm{PF}=\frac{V}{At}$$

When it comes to oil rejection, the following formula is used: one minus the ratio of permeates flow (C_p_) to oil concentrations in the feed (C_f_).

(7) $$R=\left(1-\frac{C_{p}}{C_{f}}\right)\times 100$$

The last five minutes of filtering time were used to collect all permeate samples.

### 2.6. UV Calibration Curve for Desalter Wastewater

**Figure 5:**
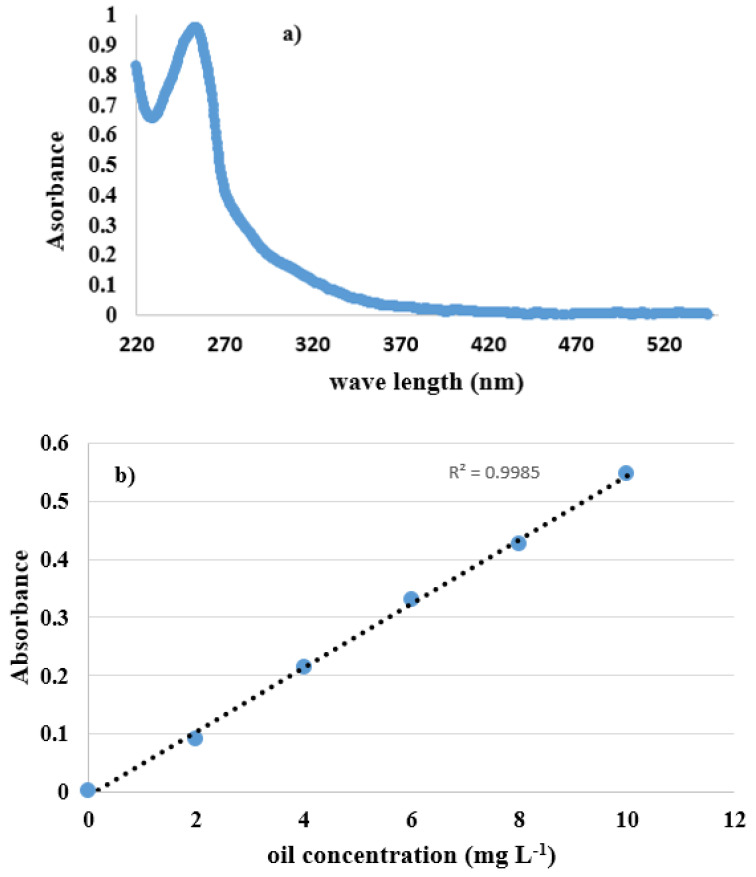
(**a**) UV-Vis absorption spectra (**b**) Calibration curve.

The emulsion has max absorption at 254 nm. The UV spectrometer (Shimadzu UV-1700, Kyoto, Japan) was equipped with a 254 nm UV (UV_254_) to detect the oil concentration in the permeation flow. Figure 5a indicates UV-visible absorption with a maximal 254-nm absorption peak. At 254 nm, maximum absorption was observed, and its corresponding λ_max_ was unaffected by the oil content. The linear calibration curve with R^2^ = 0.9985 as the coefficient of determination was obtained, as shown in Figure 5b. Salt concentration on permeate sample was determined by ionic chromatography. It was the same operating condition used for all experimental procedures throughout the experiment.

## 3. Result & Discussions

### 3.1. Membrane Characterization

#### 3.1.1. Scanning Electron Microscopy (SEM)

**Figure 6:**
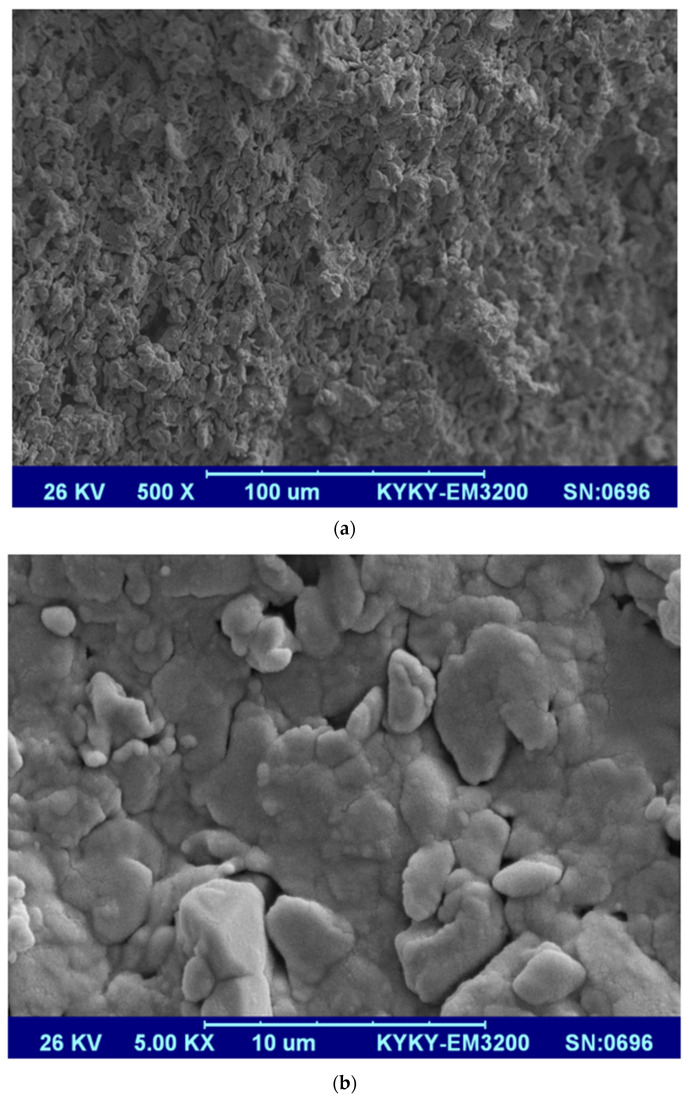

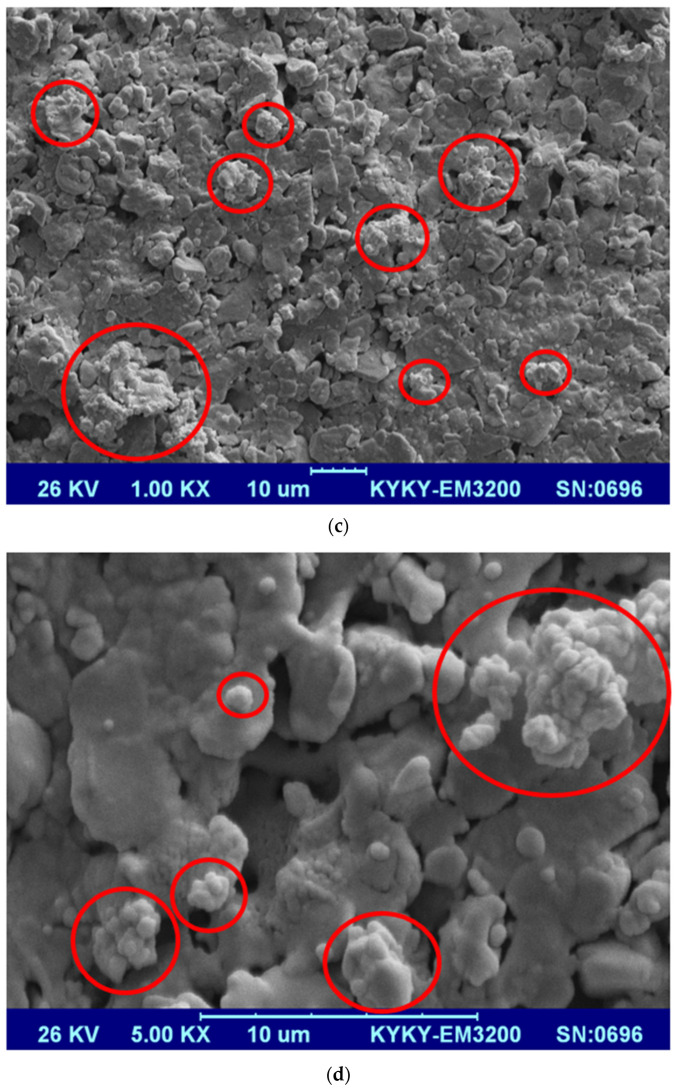

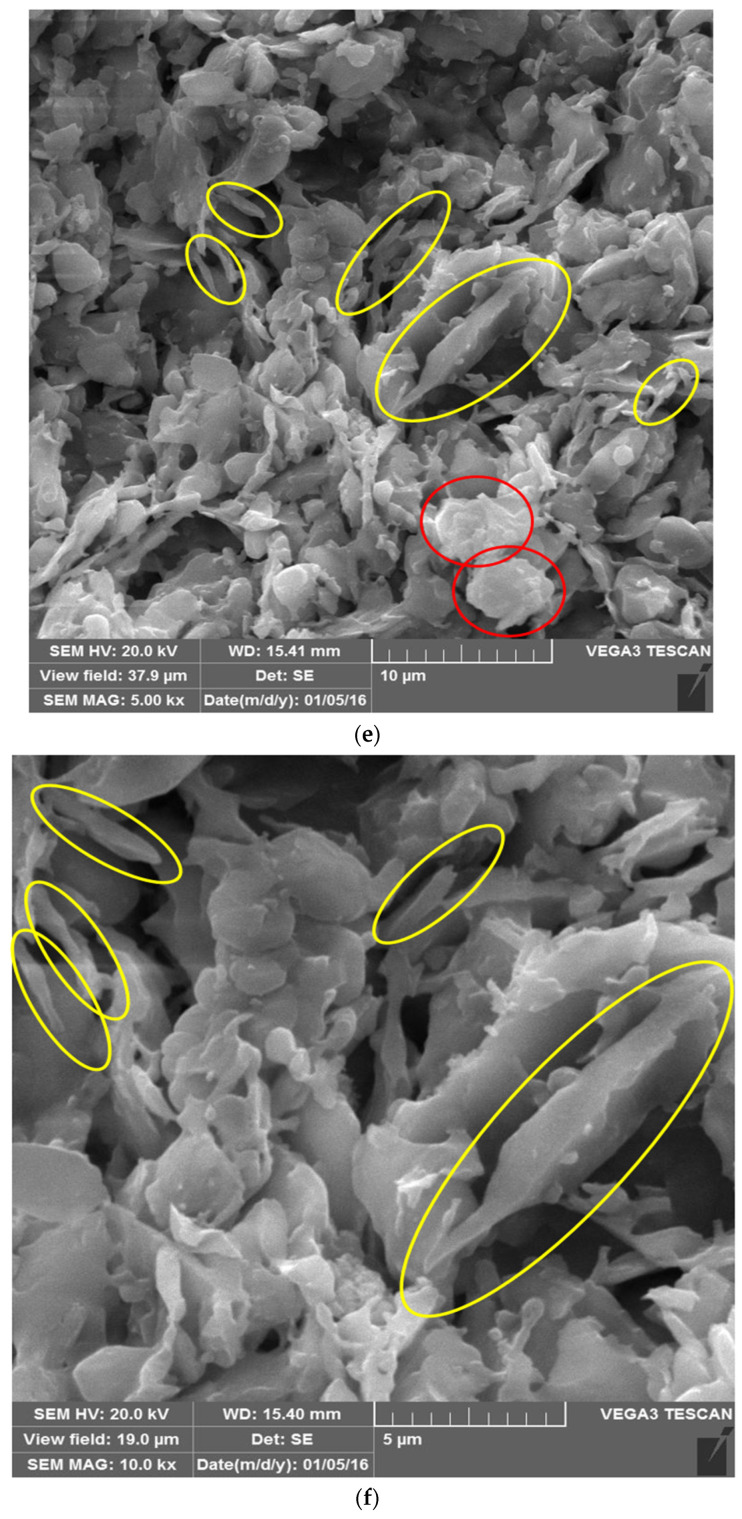

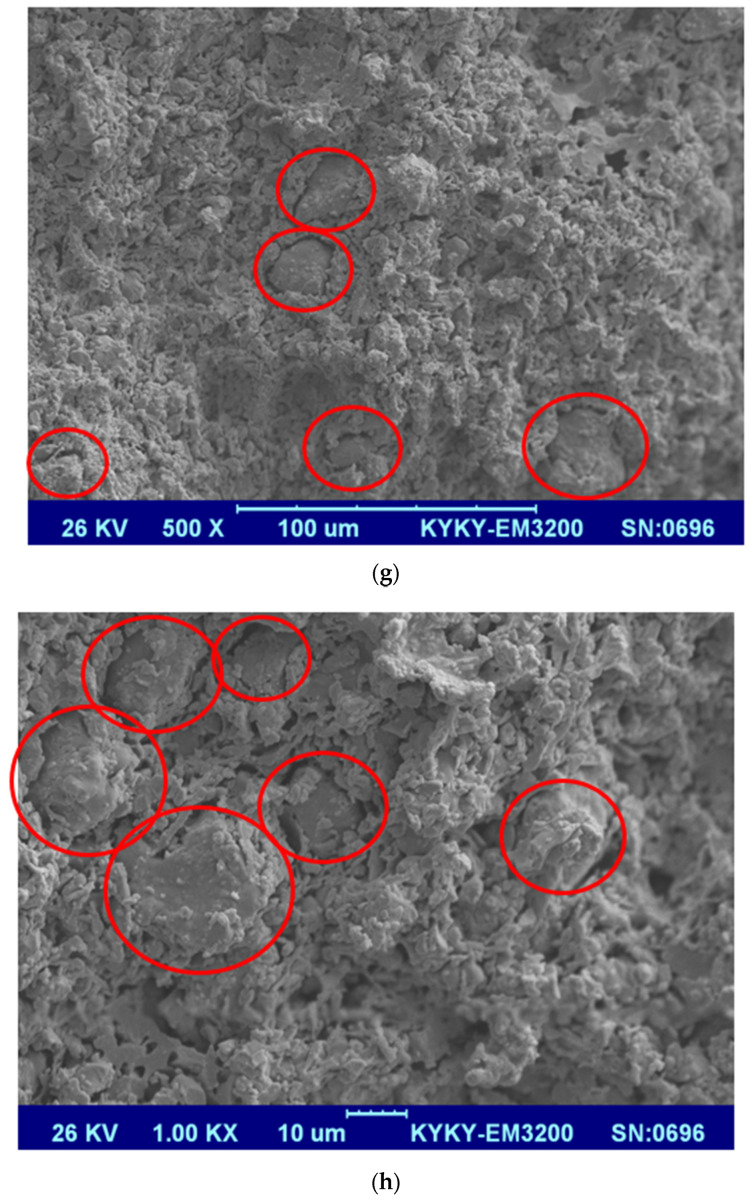
SEM image from the surface of ceramic membranes (**a**) mullite membrane with 500× zoom (**b**) mullite membrane with 5000× zoom (**c**) MA membrane with 1000× zoom (**d**) MA 50% membrane with 5000× zoom (**e**) MAZ 20% membrane with 5000× zoom (**f**) of MAZ 20% membrane with 10,000× zoom (**g**) MZ 40% membrane with 500× zoom (**h**) MZ 40% membrane with 1000× zoom.

Figure 6a,b shows SEM images from the surface of the mullite membrane with 500× and 5000× zoom. A typical homogenous and inter-locked mullite porous structure was observed for this membrane. For the MA 50%membrane, SEM micrographs with 1000× and 5000× zoom are shown in Figure 6c,d. As the red circles in these figures show, nano alpha-alumina powders are embedded well inside the MA 50%membrane’s framework. For the case of the MAZ 20%membrane, SEM images from a cross-section with the 5000× and 10,000× zoom are illustrated in Figure 6e,f. By incorporating natural zeolite and alpha-alumina powder into the MAZ 20%membrane structure, they were placed between kaolin particles (which are converted to mullite phase) and embedded effectively in the structure as the yellow (natural zeolite) and red (alpha-alumina) circles in the figure show. In the MZ 40% membrane, natural zeolite was placed in the membrane structure as the red circles in the SEM images show (Figure 6g,h) clearly. It is worth mentioning that the SEM analysis shows an asymmetric structure for all the synthesized membranes. Moreover, such membranes are aimed to be used in pre-treatment applications; therefore, they are synthesized without a selective layer. In fact, they are categorized as anisotropic asymmetric ones (not a composite membrane).

#### 3.1.2. X-ray Diffraction Analysis (XRD)

**Figure 7:**
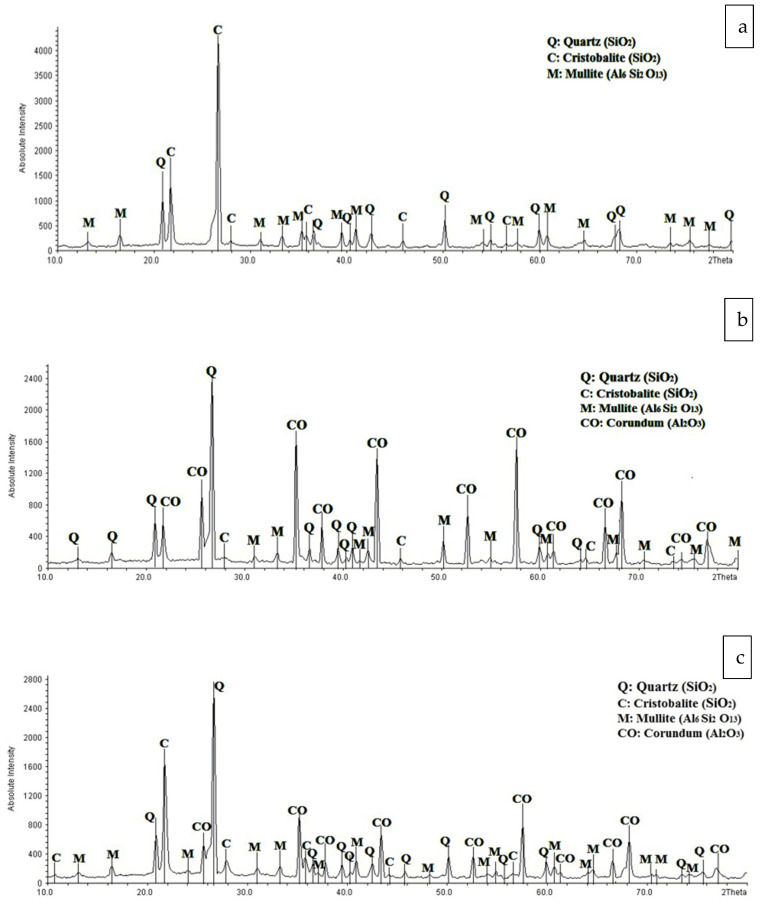

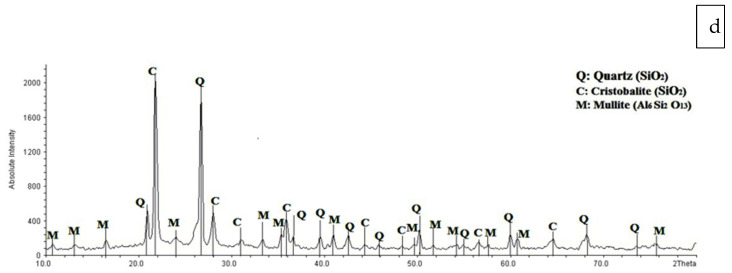
XRD pattern of prepared ceramic membranes after sintering (**a**) mullite (**b**) mullite-alumina (**c**) mullite-alumina-zeolite (**d**) mullite-zeolite.

Figure 7 depicts the XRD patterns for four different kinds of membranes. The analysis reveals that the primary and minority phases in mullite and MZ 40% membranes are mullite/quartz, and cristobalite, respectively, whereas the primary phases in the MA 50% and MAZ 20% membranes are corundum and quartz, with mullite and cristobalite as minor phases. At different temperatures and ambient pressure, the corundum is the most durable and dominant Al_2_O_3_ phase. When aluminum hydroxide and aluminum oxy-hydroxide decompose, high-temperature oxidation of alumina-forming alloys occurs, and amorphous alumina recrystallizes, other metastable Al_2_O_3_ polymorphs are generated before the most stable -Al_2_O_3_ phase is reached [45].

In the mullite and MZ 40% membranes, kaolin and natural zeolite are converted into quartz, cristobalite, and mullite phases when the sintering temperature approaches 1050 °C. During the sintering process of MA 50%and MAZ 20% membranes, alpha-alumina powder is converted to the corundum crystalline phase. As indicated in Figure 7b,c, the MA 50%membrane construction has higher absolute intensity and corundum phase production than MAZ 20% membranes due to the use of additional alpha-alumina powder.

#### 3.1.3. Void Fraction & Average Pore Radius Analysis

As depicted in Table 4, the void fraction and average pore radius of the mullite membrane are 32.6% and 0.451 µm, respectively. Adding 50 wt.% alpha-alumina powder to the structure of the MA 50% membrane increases porosity and mean pore diameter by 36.43% and 507 µm, respectively. In addition to the above findings, there is other evidence in scientific literature to support this [44]. MAZ 20% membrane exhibited the highest porosity and a mean pore diameter equal to 46.62% and 0.62 µm among all fabricated membranes since the membrane structure contains alpha-alumina as well as natural zeolite powder to enhance porosity and mean pore radius. Finally, porosity and mean pore radius for the MZ 40% membrane are reported as 39.53% and 0.556 µm, respectively.

#### 3.1.4. Mechanical Strength of the Membranes

In terms of mechanical strength, mullite, MA 50%, MAZ 20%, and MZ 40% 40% have values of 18.3, 24.6, 22.2, and 19.8 MPa. Alpha-alumina powder undergoes sintering operations, which results in the formation of the corundum crystalline phase. The corundum phase’s key properties are hardness, tensile strength, and abrasion resistance. Since more corundum phase forms in the MA membrane’s structure, it has better mechanical strength than other manufactured membranes. The mechanical strength of the MAZ 20% membrane is lower than the MA 50% membrane because the lower concentration of alpha-alumina in the MAZ 20% membrane’s structure causes less corundum phase to be formed. The incorporation of natural zeolite into the MZ membrane’s structure causes it to have more mechanical strength than a mullite membrane because more crystalline phases such as quartz are formed [41].

### 3.2. Salinity-Induced Changes in the Different Membrane’s PF

#### 3.2.1. Salinity-Induced Changes for the Mullite Membrane

**Figure 8:**
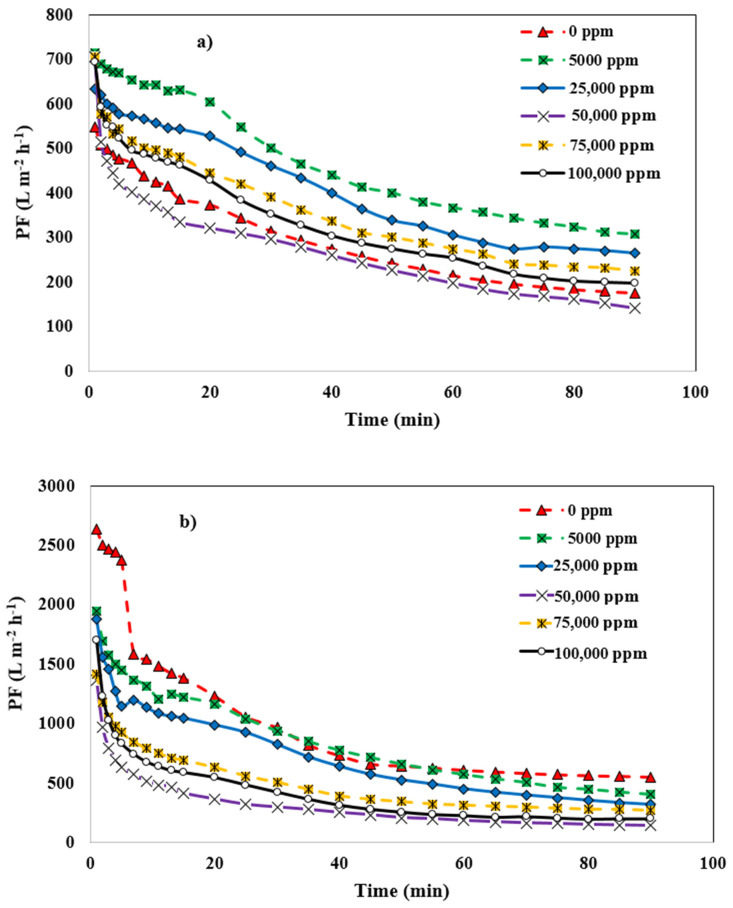

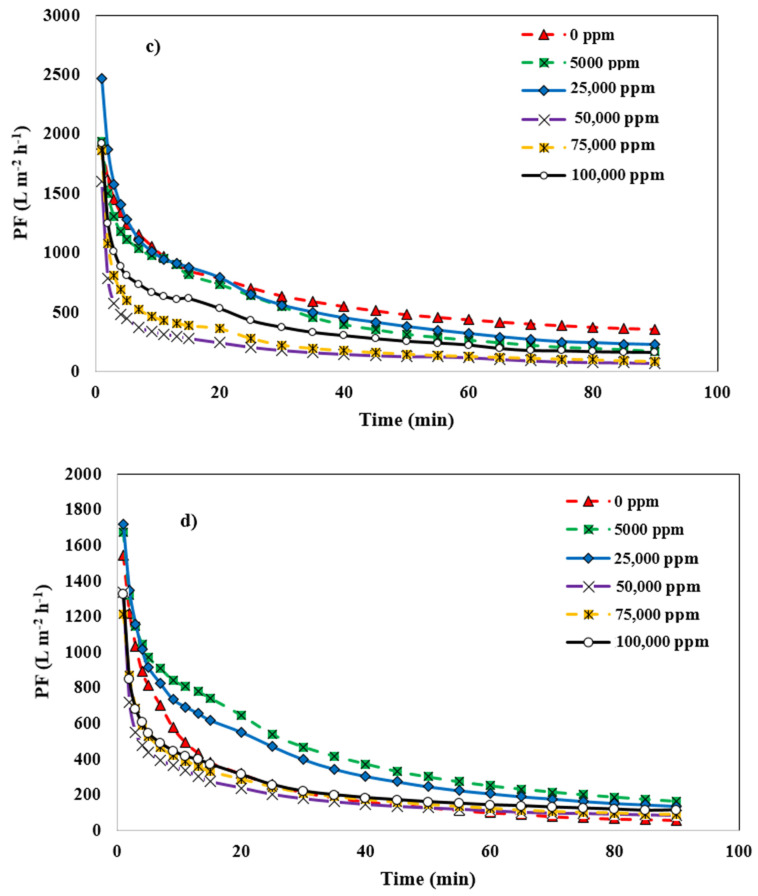
Effect of salt concentration on PF of membranes (**a**) mullite, (**b**) mullite-alumina, (**c**) mullite-alumina-zeolite, (**d**) mullite-zeolite.

Figure 8a shows the effect of increasing salt concentration in the wastewater of the desalter unit on the PF of mullite membranes. By raising the salt content to 5000 mg L^−1^, the mullite membrane’s ultimate PF is boosted from 176.09 to 307.87 L m^−2^ h^−1^. Increasing concentration of sodium chloride from 5000 mg L^−1^ to 25,000 mg L^−1^ is led to PF decline due to the higher fouling effects on the membrane surface. In the presence of 50,000 mg L^−1^ total salt concentration, PF of the mullite membrane is rapidly reduced. As filtration time increases, the salt accumulates on the membrane surface, transferring mass from the membrane surface to the bulk phase, which results in a reduction of the membrane performance in terms of PF due to reverse flow and concentration polarization. The remained salts are concentrated at the upstream membrane surface, while the concentration of transported species decreases. As the study of Tashvigh et al. [46] shows, using a 5000 mgL^−1^ of salt, and TMP of 1 bar, under a steady-state condition, increases the thickness of the concentration polarization layer along the length of the membranes.

#### 3.2.2. Salinity-Induced Changes of the MA 50% Membrane

The effect of sodium chloride enhancement concentration on the PF of the MA 50% membrane is shown in Figure 8b. Unlike the Mullite membrane, the MA 50% membrane has a lower PF when additional salt is added to the wastewater in any concentration. In fact, in lower salt concentrations (5000 & 25,000 mg L^−1^) PF increases compared to higher salt concentration (50,000 mg L^−1^). With salt concentrations ranging from 0 to 50,000 mg L^−1^, the PF of the MA 50% membrane diminishes from 549 to 139 L m^−2^ h^−1^. At lower salt concentration, due to diminished double-layer thickness surrounding emulsion droplets due to high-ionic concentrations, there is less electrostatic barrier to coalescence, which causes higher PF. The emulsion becomes viscous at greater salt concentrations, and salt crystals clog the membrane pores due to the membrane surface’s polarization caused by salt concentration; therefore, PF is decreased [44]. It’s worth noting that adding salt to the emulsion might increase the average size of the emulsion’s oil droplets by changing their surface charge, leading to the aggregation of them. PF membranes are affected by the presence of salt in two ways that are diametrically opposed. Salt crystals reduce PF, whereas larger oil droplets increase it; hence, the optimal salt concentration for PF enhancement exists [47].

#### 3.2.3. Salinity-Induced Changes for the MAZ 20% Membrane

Figure 8c shows the effect of salt addition on the PF of the MAZ 20% membrane. As the Figure plainly illustrates, when the ionic strength of the feed solution grows, membrane fouling becomes more apparent. An increment in the feed concentration led to a reduction in the membrane PF. Increasing the salt concentration boundary layer and the concentration polarization at the membrane surface contributed to this reduction. When the solute is a macromolecular compound such as a protein or oil emulsions, the solute concentration may exceed the gel concentration at the membrane-to-gel contact; at this condition, the fluidity of the solution will deteriorate. At the membrane contact, a gel layer develops, increasing resistance to the PF and hence decreasing. The PF declines until the amount of solute at the membrane interface meets the concentration of the gel, at which moment a steady state is attained [48,49]. MAZ 20% membrane PF in stable condition is decreased from 353 to 84 Lm^−2^ h^−1^ by adding a 0 to 50,000 mg L^−1^ salt concentration.

#### 3.2.4. Salinity-Induced Changes for the MZ 40% Membrane

As depicted in Figure 8d, at the lower salt concentration in the wastewater, i.e., 5000 and 25,000 mg L^−1^, PF of the MZ 40% membrane is higher than 0 mg L^−1^ salt. At lower salt concentrations, the mean radius of the oil droplets in the emulsion becomes larger because the negatively charged oil droplets are neutralized, and there is an accumulation of counter ions around them. Therefore, the formed larger flocs are settled out, and PF tends to enhance. When salt concentration is increased from 25,000 mg L^−1^ to 50,000 mg L^−1^, the lowest membrane steady state PF is 84.67 L m^−2^ h^−1^, which is in the same as other membranes are at the salt concentration of 75,000 mg L^−1^ and 100,000 mg L^−1^, PF is enhanced slightly.

### 3.3. Effect of Salt Content on Mullite-Membrane Oil Rejection

**Figure 9:**
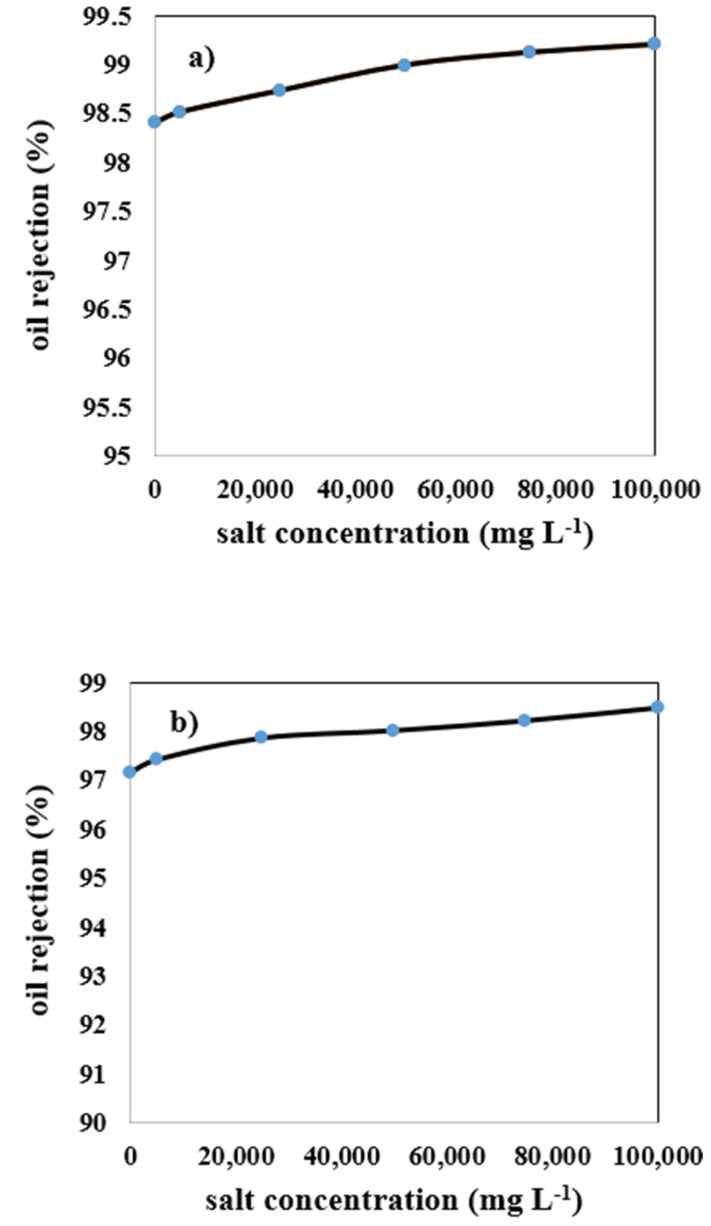

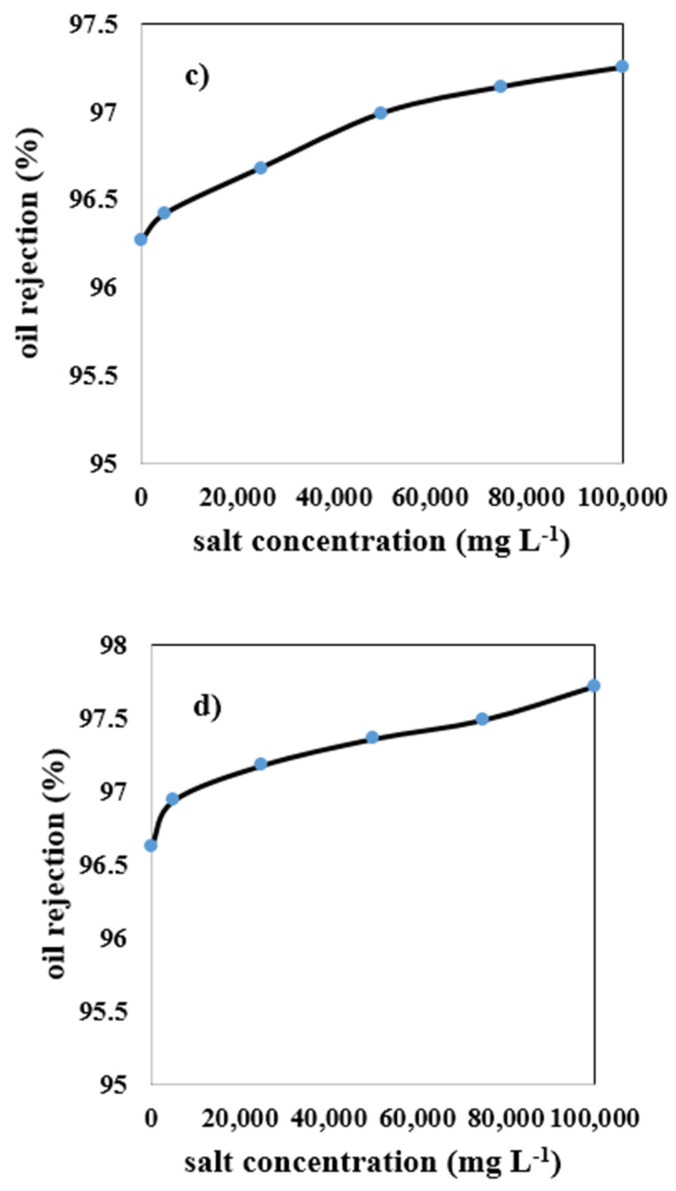
Effect of salt concentration on oil rejection of ceramic membranes (**a**) mullite, (**b**) mullite-alumina, (**c**) mullite-alumina-zeolite, (**d**) mullite-zeolite.

**Figure 10:**
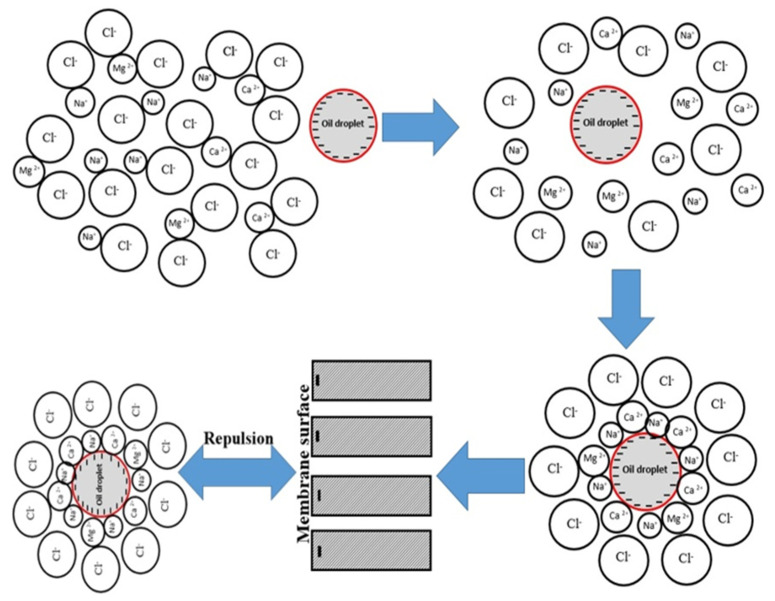
Schematic representation of effect of ionic strength and membrane surface charge on oil and salt rejection.

Figure 9a depicts the impact of increasing salt concentration on mullite membrane oil rejection. As this Figure shows, by enhancing salt concentration in the wastewater, oil rejection is increased very slightly. Salts in the intake wastewater shelter the outer electrons from the charge, lowering the membrane’s negative charge, reducing PF, and increasing oil rejection. Due to higher counter-ion concentration, higher ionic strength leads to a minor increase in charge density while simultaneously sheltering the charges, which reduces electrostatic attraction between organic molecules. As a result, there will be a more significant drop in PF, and high oil rejection occurs at higher ionic strength [50]. Oil rejection increased from 98.4% to 99.2% for this membrane by employing 100,000 mg L^−1^ salt concentration. The double layer of a charged solute or a charged membrane surface may be compressed by increasing the ionic strength of the solution; because of this, there will be less of an electrostatic connection between them. This behavior was consistently observed when either CaCl_2_ or NaCl was used to increase the feed solution ionic strength. An increase in the feed solution ionic strength caused the membrane surface and charged solute’s electrical double layer to compress [51]. Another explanation for the increase in oil rejection by ionic strength may be attributed to larger oil droplets due to higher ionic strength. By increasing ionic strength, cations accumulate around negatively charged oil droplets, and the mean diameter size of the micelle becomes larger; therefore, oil rejection is increased. A schematic representation of this phenomenon is shown in Figure 10.

#### 3.3.1. Effect of Salt Content for MA 50% Membrane

For the MA 50% membrane, similar to the reasons mentioned in Section 3.3 regarding the Mullite membrane, increasing the salt concentration in the wastewater led to a slight upward trend in the performance of oil rejection. As illustrated in Figure 9b, with an increment in the salt content from 0 to 100,000 mg L^−1^, oil rejection is enhanced from 97.1% to 98.4%, respectively. The MA 50% membrane has lower oil rejection results than the mullite membrane because of its higher porosity and mean pore size, which leads to a higher PF and lower oil rejection.

#### 3.3.2. Effect of Salt Content for MAZ 20% Membrane

The effect of increasing salt concentration in the wastewater on the oil rejection of the MAZ 20%membrane is shown in Figure 9c. Without adding salt to the wastewater (0 mg L^−1^), oil rejection is reported at 96.2%. By enhancing salt concentration from 0 to 100,000 mg L^−1^, oil rejection is boosted from 96.2% to 97.2%, respectively. As the MAZ 20%membrane has the largest mean pore size among the other membranes, micelles with a larger size can get through the porous medium and reduce the amount of oil rejected.

#### 3.3.3. The Impact of Salt Content for MZ 40% Membrane

According to Figure 9d, for the MZ 40%membrane, like other membranes, the increase of salt concentration from 0 to 100,000 mg L^−1^ in the synthetic wastewater resulted in an increase from 96.6% to 97.7% oil rejection. The reason for increasing oil rejection with salt concentration is similar to the reasons mentioned in Section 3.3 for a mullite membrane, but it must be noted that MZ 40% membrane has moderate mean pore size and porosity among the other membranes; therefore, the percentage of oil rejection obtained by this type of membrane is higher and lower than MAZ 20% and mullite membranes respectively.

### 3.4. Effect of Salt Concentration in the Wastewater on the Ion Rejection by Four Types of Membranes

**Figure 11:**
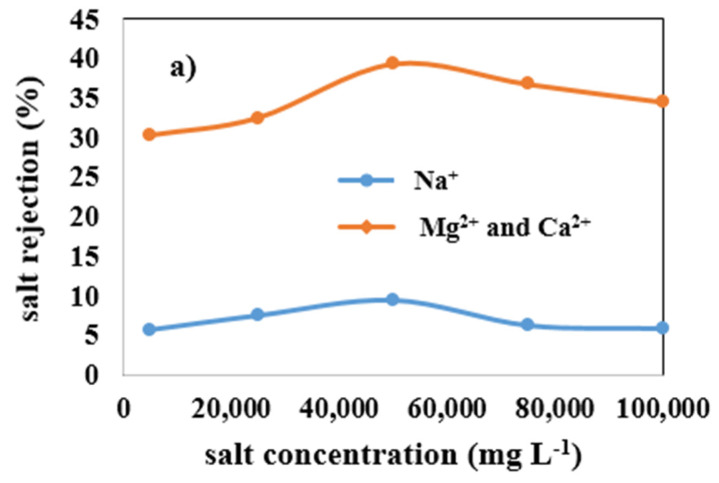

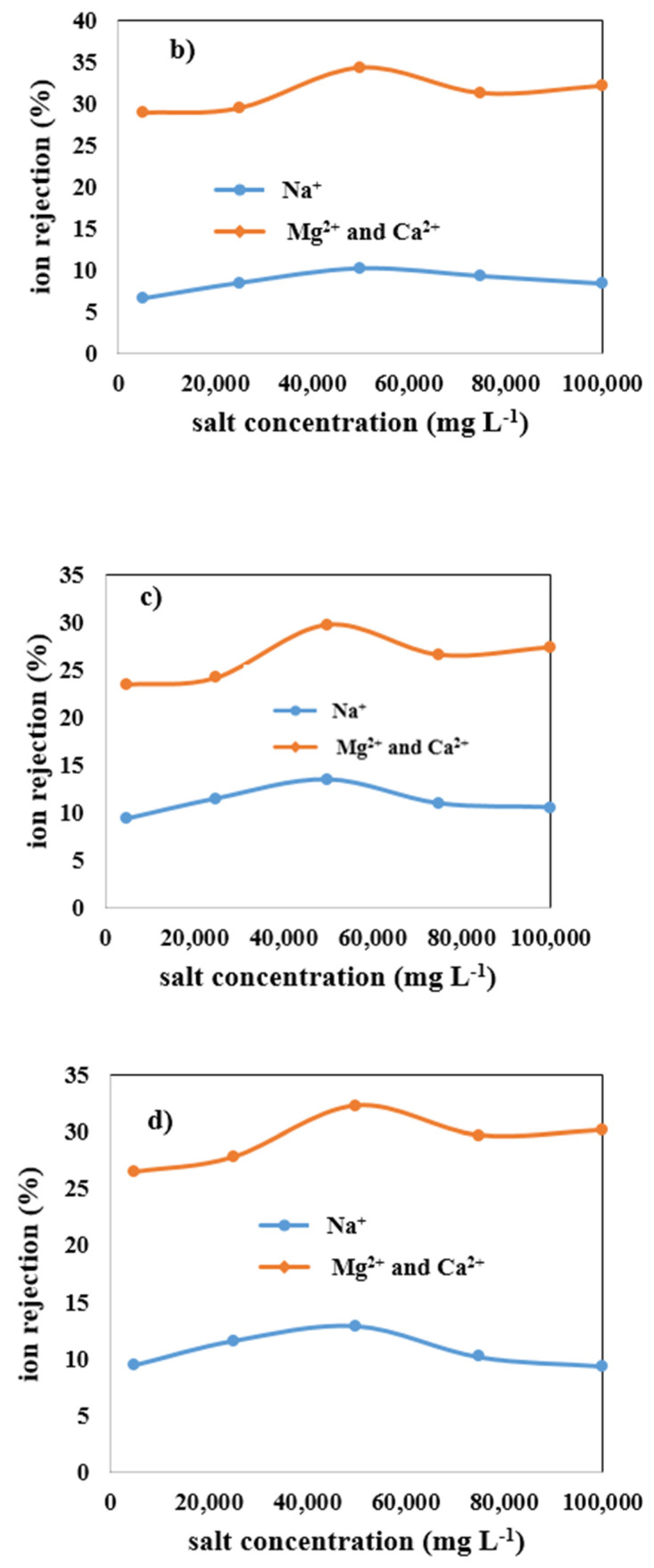
Effect of increasing salt concentration in wastewater on monovalent (Na^+^) and multivalent (Mg^2+^ and Ca^2^+) ions rejection of membranes, (**a**) mullite, (**b**) mullite-alumina, (**c**) mullite-alumina-zeolite, (**d**) mullite-zeolite.

**Figure 12:**
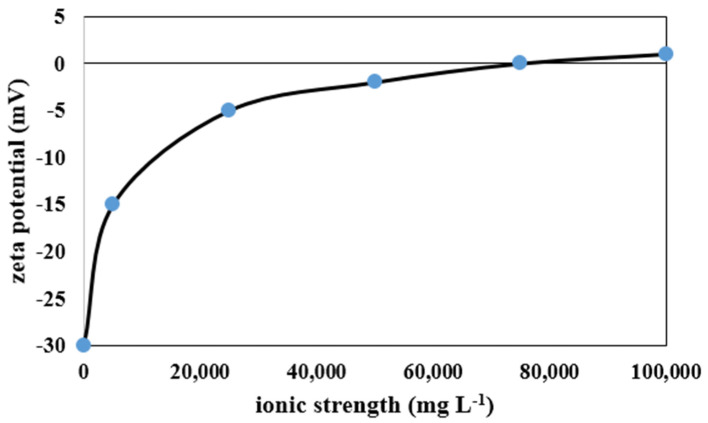
Effect of ionic strength on zeta potential of synthetic wastewater of desalter unit.

Figure 11 shows the effect of enhancing salt concentration in wastewater on the monovalent (Na^+^) and multivalent (Mg^2+^ and Ca^2+^) ions rejection by mullite (Figure 11a), MA 50% (Figure 11b), MAZ 20% (Figure 11c), and MZ 40% (Figure 11d) membranes. By the addition of total salts up to 50,000 mg L^−1^ in the wastewater, ion rejection by all types of membranes is increased. The highest monovalent and multivalent ions rejection were observed when 50,000 mg L^−1^ salt concentration was added to the wastewater for all ceramic membranes; as mentioned before, the lowest PF of all membranes occurred through this concentration of salt. For the mullite membrane, by employing 50,000 mg L^−1^ salt in the feed, monovalent and multivalent ion rejection increased up to 9.5% and 39.35%, respectively. Ion rejection decreased slightly when the salt concentration was raised from 50,000 to 100,000 mg L^−1^. In fact, there is an optimum salt concentration for increasing PF, oil, and ion rejection. Table 5 presents the variation of pH values by increasing salt concentration in the wastewater. The membrane’s negative surface charge is raised by boosting pH, leading to an increase in electrostatic repulsion between a negatively charged solute and the membrane surface. The increase of ions such as Na^+^, K^+^, Ca^2+^, and Mg^2+^ in the feed wastewater resulted in a reduction in the negatively charged zeta potential of a membrane owning to the neutralization of oil droplets with a negative charge. The zeta potential of synthetic oily wastewater of desalter unit versus ionic strength is shown in Figure 12. In the wastewater with low electric field strength in a solution, the zeta potential is negative and decreases with increasing ionic strength. As a result of ionic strength increase, the zeta potential sign changes. As a result, these ions are deposited on the surface of the membrane and lead to a decline in PF. The deposited layer on the membrane surface prevents ions from passing through the membrane pores; thus, salt and oil rejection are increased [52].

## 4. Conclusions

In this study, the effects of increasing salt concentration (0, 5000, 25,000, 50,000, and 100,000 mg L^−1^), along with the effect that types of ceramic membranes had on the membrane performance in terms of PF, oil, and ion rejection are studied. For this purpose, four different kinds of novel and inexpensive microfiltration membranes made of ceramic (mullite, MA 50%, MAZ 20%, and MZ 40%) were fabricated using kaolin clay, alpha-alumina powder, and natural zeolite powder via extrusion method. Fabricated membranes were characterized by using characterization techniques such as SEM, XRD, mean pore size, porosity measurement, and the ability to withstand mechanical stress in order to ensure correct preparation. Characterization results showed that all types of membranes were prepared in the correct way, desired crystalline phases were formed, and desired porous morphology was obtained. The results of membrane application for desalter wastewater treatment showed that PF of Mullite and Mullite-zeolite membranes was increased with low concentrations (5000 mg L^−1^ and 25,000 mg L^−1^) of salts compared to 0 mg/L of salt. For Mullite-alumina and Mullite-alumina-Zeolite membranes, the lower salt concentrations showed a higher flux compared to the higher ones, and the flux values were roughly close to the flux without salt addition. At a higher concentration of salt, the PF of membranes decreased due to the concentration polarization and high fouling effects. By increasing salt concentration in the wastewater, oil and ion rejection in all membranes increased slightly. Oil rejection was reported to be between 96.2% and 99.2%. Monovalent and multivalent ions rejection was about 5% to 13% and 23% to 40%, respectively.

## Figures and Tables

**Table 1:** Salt concentration in desalting wastewater for Dehluran oil field, Iran [3].

Ion	Concentration (mg L^−1^)	Ion	Concentration (mg L^−1^)
Na^+^	65,633	Fe^2+^	12.5
Ca^2+^	8350	Cl^−^	118,925
Mg^2+^	1000	HCO_3_^−^	153
SO_4_^2−^	216		

**Table 2:** Analyses of the chemical composition of kaolin clay and natural zeolite used to fabricate ceramic membranes.

Component.	Percent (%)		Phases	Percent (%)
	Kaolin	Natural zeolite	kaolin	kaolin
SiO_2_	61.62	68.5	Kaolinite	64
TiO_2_	0.4	-		
Al_2_O_3_	24–25	11	Illite	2.4
Fe_2_O_3_	0.45–0.65	0.2–0.9		
K_2_O	0.4	4.4	Quartz	27
Na_2_O	0.5	3.8		
L.O.I	9.5–10	10–12	Feldespare	6.6
Total	100	100		100

**Table 3:** Analyses of crude oil’s properties, both physical and chemical used for preparing synthetic wastewater of desalter unit.

Value	Unit	Specification	Value	Unit	Specification
4.83	psi	R.V.P	0.8750	@ 15.6 °C	Sp Gr
3.60	wt.%	Asphaltene Content	30.21	-	API Gravity
6.06	wt.%	Wax	1.62	wt.%	Sulfur Content
56	oC	D.Me.Point of Wax	<1.0	wt. mg L^−1^	H2S
5.19	wt.%	C.C.R.	0.21	wt.%	Total Nitrogen
0.025	wt.%	Ash Content	0.05	vol.%	B.S & W
0.05	mgKOH/g	Acidity	0.05	vol.%	Water Content
29	mg L^−1^	Nickel	8	PTB	Salt Content
105	mg L^−1^	Vanadium	28.27	@10 °C cST	Kin Vis
2.6	mg L^−1^	Iron	16.82	@20 °C cST	Kin Vis
<1	mg L^−1^	Lead	8.410	@40 °C cST	Kin Vis
10	mg L^−1^	Sodium	−18	°C	Pour Point

**Table 4:** Materials, porosity, and average pore radius for all ceramic membranes created.

Number	Membrane	Kaolin%	Alumina%	Zeolite%	Porosity%	Mean Pore Radius (µm)
1	Mullite	100	0	0	32.6	0.451
2	Mullite-alumina	50	50	0	36.43	0.507
3	Mullite-alumina-zeolite	50	30	20	46.62	0.62
4	Mullite-zeolite	60	0	40	39.53	0.556

**Table 5:** Variation of pH at different concentrations of salt in the desalter wastewater.

Salt concentration (mg L^−1^)	5000	25,000	50,000	75,000	100,000
pH	7.6 ± 0.1	7.3 ± 0.1	7 ± 0.1	6.8 ± 0.1	6.7 ± 0.1

## Data Availability

Not applicable.

## Nomenclature

**Notation**	
A	Effectiveness area of membrane (m^2^)
CFV	Cross flow velocity (m·s^−1^)
d_average_	Average droplet size of oil in the emulsion (µm)
di	Each oil droplet diameter size in the emulsion (µm)
L	Membrane thickness (m)
n	Dimensionless
PF	Permeation flux (L m^−2^ h^−1^)
r_m_	Mean pore radius of membrane (µm)
TMP	Transmembrane pressure (bar)
PTB	Pound per thousand barrels
T	Temperature (°C)
t	Time (minutes)
V	Volume of permeate (Liter)
V_M_	Volume of the membrane (m^3^)
W_1_	Mass of dry membrane (gr)
W_2_	Soaked membrane’s mass (gr)
**Greek Letters**	
ρm	Water density at the experiment’s temperature (kg m^−3^)
ε	Porosity of the membrane (dimensionless)
μ	Water viscosity at the operating temperature (Pa·s)
